# Skin Elasticity of the Face: Demographic and Lifestyle Intricacies

**DOI:** 10.1111/srt.70209

**Published:** 2025-08-05

**Authors:** Gitanjali Petkar, Adaam Domun, Nusayha Sokeechand, Muzzammil Hosenally

**Affiliations:** ^1^ Centre International de Développement Pharmaceutique (CIDP) Phoenix Mauritius; ^2^ Department of Economics and Statistics University of Mauritius Réduit Mauritius

**Keywords:** Cutometer, demography, elasticity, lifestyle

## Abstract

**Background:**

The assessment of improvements in facial skin features using instrumental measurements is often challenging. This is also true for the assessment of signs of ageing within various populations. For the purpose of clinical studies, there is a need to document the values that could be generated using such instrumental measurement. This paper, thus, investigates the effect of demographic profile and lifestyle on facial elasticity using an elasticity meter on a diverse and multiethnic population.

**Materials and Methods:**

This one‐day clinical study involved the participation of 300 participants for demographic profiling and self‐reported lifestyle (after consenting). Skin elasticity measurements were taken using an elasticity meter in controlled conditions. Generated data included both absolute and relative parameters. These were tabulated using simple descriptive statistics, by demographic profile and lifestyle. Inferential statistical analyses were applied to assess for significant differences among groups.

**Results:**

Benchmark instrumental values are reported in the form of central tendencies and dispersions. Gender, age, phototype, ethnicity, occupation and transportation methods revealed salient differences in terms of skin elasticity. On the other hand, only non‐significant trends were noted for other demographic characteristics and lifestyle behaviours investigated.

**Conclusion:**

The findings propose some benchmark values that could be used to inform the design of future clinical trials. This includes sample size calculations, taking into consideration the panels being studied. There is a need to take into consideration the demographic and lifestyle background of the proposed panel when designing a study aimed at improving facial skin ageing features. These factors may have an influence on the response, thus confounding the study results and conclusions. Future longitudinal studies looking at long‐term behavioural influences are required to better understand the dynamics of skin elasticity.

## 1 Introduction and Background

The skin, the largest organ of the body, plays a crucial role in barrier function and sensorineural and psychosocial perception of an individual [1]. Skin ageing is a distinct phenomenon characterised by a loss of elasticity, hydration, firmness, reduction in the epidermal thickness and collagen content, manifesting as sagging of skin, appearance of wrinkles and pigmentation [2, 3]. Various mechanisms are postulated to accelerate the ageing process, namely, collagen degradation, lower elastin levels, reduced blood flow to epidermis, reactive oxygen species (ROS) and advanced glycation end products [4, 5, 6, 7, 8, 9, 10, 11]. Reduced collagen synthesis due to ageing, coupled with degraded elastin arising from matrix metalloproteinases and other enzymes, contributes to skin weakening. Similarly, imbalances in terms of ROS production contribute to the deterioration of desirable facial skin features such as elasticity.

Multiple entities such as chronological ageing, photoageing, environmental pollution and lifestyle factors including smoking, unhealthy dietary habits, lack of exercise, sleep and stress are considered to act synergistically to influence its course [12, 13, 14, 15]. Environmental influences on skin ageing properties also draw from regional differences observed on the body in terms of skin biomechanical properties, specifically elasticity and viscoelasticity [16, 17].

The skin biomechanical properties, mostly elasticity and viscoelasticity, have proved to be useful biomarkers of skin ageing. These skin properties can be measured using a Cutometer, which is a non‐invasive suction‐based instrument providing for the possibility to quantify subtle differences and changes in skin. The derived parameters, ranging from absolute ones to relative ones, are widely documented in the literature. In the study conducted on Korean women [16], Kim et al. demonstrated a strong correlation between relative parameters of skin elasticity, *R*2 (*U*a/*U*f) and *R*7 (*U*r/*U*f), and changes related to skin ageing, using a Cutometer.

The effect of various demographic characteristics and lifestyle on skin ageing is still unclear or confounded, requiring further investigation. Lifestyle habits such as weight and cigarette consumption have been shown to negatively impact the signs of ageing, whereas the influence of alcohol consumption was positive [15]. In a narrative review, Oizumi et al. [18] argued that exercise can potentially enhance skin function retention, but care should be taken with respect to the type of exercise for skin ageing considerations. The effect of BMI also revealed contradictory results; in one study [19] the elasticity of participants with lower BMI was unexpectedly less than that in those with higher BMI, whereas in another study [20], no such patterns were discerned. The literature is also unclear on the effect of gender on skin ageing properties, varying to some degree by body site, and while some tending to indicate slower signs of ageing for men [21, 22, 23].

Thus, this study seeks to document the effect of common demographic indicators and selected behavioural lifestyle on skin Cutometer measurements. It aims at contributing to a further understanding of the intricacies underlying the derived parameters, discerning patterns between subjects from various sociodemographic backgrounds and lifestyle. The objective is to present findings in a descriptive manner to support informed clinical trial designs, which take into consideration factors that could possibly influence the response, and clearer interpretation of the results generated thereof.

## 2 Materials and Methods

### 2.1 Study Design and Population

This was a monocentric, open and intraindividual clinical study conducted at CIDP LTEE in Mauritius (BioPark, SOCOTA Phoenicia, Phoenix, Mauritius) between 25 March 2024 and 06 June 2024. The study aimed to recruit 300 male and female subjects. To be included, participants were required to be between 18 and 65 years of age, and agreeing to refrain from applying cosmetic products on the day of the visit. The exclusion criteria restricted the enrolment of subjects with systemic disorder or skin diseases, immunological disorder, any history of medical/surgical events that according to the investigator could compromise the safety of the subject or affect the outcome of the study, and subjects who were pregnant or lactating. Subject selection was convenience‐based; prospective candidates for upcoming clinical trials at the clinical research organisation were proposed to embark on this study as well as part of their visit to the clinical research organisation.

### 2.2 Skin Elasticity Measurements

Measurements were taken in comfortable and controlled conditions (22 ± 2°C and 50 ± 10% RH). Mechanical properties of the skin were determined with a non‐invasive suction‐based skin elasticity meter, namely, the Cutometer Dual MPA 580 (Courage Khazaka Electronic GmbH, Cologne, Germany). A 2‐mm diameter measuring probe was used, which applied a constant suction of 300 mbar. Measurements were made on a single site on the cheek generating a graph depicting the absolute parameters, namely, immediate deformation (*U*e), final deformation (*U*f), delayed distension (*U*v), immediate retraction (*U*r) and total recovery (*U*a). Relative parameters are mathematically computed, namely, *U*a/*U*f, defined as the ability of the skin to return to its original position after deformation, *U*r/*U*f, referred to as the biological elasticity, *U*r/*U*e, defined as the pure elasticity and *U*v/*U*e, which is the ratio of viscoelastic to elastic extension.

### 2.3 Lifestyle Questionnaire

Participants were asked to complete a questionnaire under the supervision of a clinical staff member. The questionnaire is composed of various eight (8) sections described below.

**Table:** 

Construct/section	Items	Scoring
Demography	Gender, age, phototype, ethnicity, skin type on face/body, BMI	None
Physical activity *based on readily available Health Risk Assessment Questionnaire* [24]	I engage in moderate physical activity outside of work at least 20–30 min at least 5 days of the week. My physical activity includes stretching, aerobic and strength conditioning. I use alternative modes of transportation whenever possible to and from various locations (i.e stairs instead of elevator, biking or walking instead of driving). I take the health benefits of physical activities and their lasting impact seriously. I enjoy sedentary activities rather than physical activities.	Each item scored on a 5‐point scale. Scores aggregated to form an overall ‘physical activity’ score.
Nutrition *based on readily available Health Risk Assessment Questionnaire* [24]	I eat at least five servings of fruits and vegetables every day (one serving equals one half cup). I eat at fast food restaurants less than three times per week. I include foods that are high in fibre in my diet on a daily basis (i.e. whole grain breads and cereals, beans, etc.). I maintain a healthy weight within the recommendations specified by a health care professional. I avoid eating foods that are high in fat such as whole milk, fried foods, fatty meats, etc.	Each item scored on a 5‐point scale. Scores aggregated to form an overall ‘nutrition’ score.
General health *based on readily available Health Risk Assessment Questionnaire* [24]	I avoid the use of tobacco products (cigarettes, smokeless tobacco, cigars and pipes) and limit myself to five drinks of alcohol a week (beer, liquor, wine). I examine my breasts or testes on a monthly basis. I protect my skin from sun damage by using sunscreen, wearing hats and/or avoiding tanning booths and sunlamps. I visit my dentist every 6 months for regular check‐ups. I see my physician for routine check‐ups, health screenings and disease prevention.	Each item scored on a 5‐point scale. Scores aggregated to form an overall ‘general health’ score.
Fatigue inventory *adapted version of the Multidimensional Fatigue Inventory (MFI)* [25]	I feel tired. I feel physically exhausted. My thoughts easily get confused. I feel no desire to do anything. I do not feel like doing anything.	Each item scored on a 5‐point scale (1: That is true to 5: No that is not true). Scores aggregated to form an overall ‘fatigue’ score and categorised as ‘Low fatigue’ and ‘High fatigue’.
Sleep quality *based on readily available questionnaire* [26]	During the past 7 days, how would you rate your sleep quality overall?	11‐point scale. The categories created were ‘poor’, ‘fair’ and ‘good’.
Occupation *International Classification of Occupation* [27]	What is your current occupation?	None (open ended)
Mode of travel	What is your primary mode of transportation for going to work/school/attend common daily activities?	None

### 2.4 Study Procedure

Participants were instructed to refrain from using any cosmetics, including moisturisers, on the day before the examination. Demographic data (including gender, age, type of skin [dry/normal], Fitzpatrick phototype [I–VI], ethnicity, BMI and occupation) were collected and verification of inclusion criteria and non‐inclusion criteria along with collection of previous concomitant medication and information about subject's medical history by the investigator was done. Subjects were then asked to fill in a lifestyle questionnaire before taking Cutometer measurement in the face, more precisely on the cheeks.

### 2.5 Ethical Considerations

The study (Protocol reference 2324CMCL085) was approved by an independent ethics committee (Ebène, Mauritius, 04 March 2024). Written informed consent was obtained from all subjects in accordance with the latest revision of the Declaration of Helsinki (5). Furthermore, the study was conducted in line with the general principles of Good Clinical Practice (GCP) and its amendments.

### 2.6 Statistical Analysis

Quantitative data were expressed using the mean as measure of central tendency and standard deviation as dispersion. Qualitative data were presented as counts and percentages. Because the normality of the data was generally not violated, group comparisons involving two levels were conducted using the independent samples *t*‐test, whereas a one‐way ANOVA was used whenever the comparison of more than two levels was involved. All statistical analyses were conducted at 5% level of significance.

The following parameters were investigated:

**Table:** 

Parameter	Interpretation
*U*e	Immediate deformation (elastic extension)
*U*v	Intermediate deformation (viscoelastic deformation)
*U*f	Final deformation (*U*e + *U*v)
*U*r	Immediate recovery
*U*a	Final recovery
*U*a/*U*f	Overall elasticity
*U*r/*U*e	Pure elasticity
*U*v/*U*e	The ratio of viscoelastic to elastic extension
*U*r/*U*f	The ratio of elastic recovery to the total deformation

## 3 Results

The panel concerned for this analysis comprises a total of 298 volunteers; their demographic characteristics are summarised in Table 1 below. Briefly described, female volunteers accounted for the majority of the panel (83.6%, *n* = 249), and the distribution of age was fairly spread across the various categories. The population analysed consists mostly of subjects of phototype IV–VI (89%, *n* = 265), with more than half of the volunteers categorised as mixed race (56.7%, *n* = 169), followed by Indian (28.5%, *n* = 85) and Asian (12.1%, *n* = 36). Caucasians accounted for only 2.7% (*n* = 8) of the panel.

**TABLE 1:** Demographics.

		*n*	%
Gender	Male	49	16.4
	Female	249	83.6
Age	18–25	48	16.1
	26–35	93	31.2
	36–45	85	28.5
	46–55	43	14.4
	56+	29	9.7
Phototype	II+III	33	11.1
	IV	142	47.7
	V+VI	123	41.3
Ethnicity	Caucasian	8	2.7
	Indian	85	28.5
	Asian	36	12.1
	Mixed Race	169	56.7
Skin type on the face	Dry	43	14.4
	Non‐dry	255	85.6
Skin type on the body	Dry	97	32.6
	Non‐dry	201	67.4
BMI	Healthy/underweight	129	43.3
	Overweight	102	34.2
	Obesity	67	22.5
Occupation	Professionals	48	16.1
	Technicians and associate professionals	44	14.8
	Managers	8	2.7
	Clerical support workers	26	8.7
	Elementary occupations	15	5.0
	Service and sales workers	48	16.1
	Skilled agricultural/Plant operators, etc.	5	1.7
	Craft and related trades workers	10	3.4
	Self‐employed	15	5.0
	Housewife	47	15.8
	Student	21	7.0
	Unemployed/Retired/Unknown	11	3.7

### 3.1 Demography and Cutometer Measurements

The results of the absolute and relative parameters of interest, by demographic profile are detailed in Tables 2 and 3, respectively.

**TABLE 2:** Results of the absolute Cutometer parameters, by demographic profile.

*N* = 298		*U*e			*U*v			*U*f			*U*r			*U*a		
		Mean	SD	*p* value	Mean	SD	*p* value	Mean	SD	*p* value	Mean	SD	*p* value	Mean	SD	*p* value
Gender	Male (*n* = 49)	0.209	0.091	<0.001	0.083	0.020	0.804	0.291	0.098	<0.001	0.162	0.085	<0.001	0.232	0.101	<0.001
	Female (*n* = 249)	0.276	0.082		0.084	0.034		0.360	0.089		0.225	0.086		0.297	0.091	
Age	18–25 (*n* = 48)	0.290	0.089	0.001	0.080	0.017	0.271	0.370	0.097	0.013	0.273	0.086	<0.001	0.332	0.099	<0.001
	26–35 (*n* = 93)	0.285	0.087		0.082	0.020		0.367	0.094		0.239	0.080		0.309	0.092	
	36–45 (*n* = 85)	0.246	0.087		0.090	0.049		0.336	0.090		0.204	0.081		0.274	0.090	
	46–55 (*n* = 43)	0.246	0.076		0.081	0.024		0.327	0.091		0.167	0.074		0.253	0.084	
	56+ (*n* = 29)	0.240	0.078		0.079	0.022		0.319	0.091		0.143	0.068		0.228	0.079	
Phototype	II+III (*n* = 33)	0.232	0.058	0.001	0.080	0.017	0.054	0.312	0.065	<0.001	0.180	0.063	<0.001	0.261	0.059	<0.001
	IV (*n* = 142)	0.254	0.083		0.080	0.033		0.334	0.088		0.203	0.089		0.269	0.093	
	V+VI (*n* = 123)	0.286	0.093		0.089	0.033		0.375	0.101		0.237	0.089		0.313	0.101	
Ethnicity	Caucasian (*n* = 8)	0.241	0.080	<0.001	0.076	0.022	0.003	0.317	0.092	<0.001	0.161	0.042	<0.001	0.259	0.068	<0.001
	Indian (*n* = 85)	0.249	0.080		0.076	0.021		0.325	0.088		0.200	0.085		0.261	0.090	
	Asian (*n* = 36)	0.221	0.059		0.076	0.016		0.296	0.063		0.177	0.075		0.250	0.070	
	Mixed race (*n* = 169)	0.283	0.091		0.090	0.038		0.373	0.096		0.232	0.091		0.309	0.099	
Skin type on the face	Dry (*n* = 43)	0.244	0.085	0.096	0.086	0.048	0.651	0.330	0.084	0.166	0.195	0.088	0.109	0.266	0.094	0.122
	Non‐dry (*n* = 255)	0.268	0.087		0.083	0.028		0.352	0.095		0.218	0.088		0.290	0.096	
Skin type on the body	Dry (*n* = 97)	0.266	0.099	0.893	0.089	0.036	0.065	0.354	0.106	0.480	0.215	0.090	0.997	0.289	0.103	0.720
	Non‐dry (*n* = 201)	0.264	0.081		0.081	0.029		0.346	0.088		0.215	0.088		0.285	0.092	
BMI	Healthy/underweight (*n* = 129)	0.266	0.083	0.962	0.081	0.033	0.095	0.348	0.089	0.774	0.220	0.087	0.634	0.290	0.093	0.813
	Overweight (*n* = 102)	0.263	0.087		0.082	0.022		0.345	0.098		0.208	0.085		0.282	0.094	
	Obese (*n* = 67)	0.264	0.095		0.091	0.040		0.355	0.098		0.215	0.097		0.288	0.103	
Occupation	Professionals (*n* = 48)	0.259	0.089	0.213	0.079	0.025	0.438	0.338	0.104	0.292	0.207	0.089	0.027	0.280	0.100	0.087
	Technicians and associate professionals (*n* = 44)	0.265	0.078		0.084	0.018		0.349	0.082		0.212	0.075		0.278	0.081	
	Managers (*n* = 8)	0.268	0.074		0.084	0.017		0.352	0.088		0.217	0.078		0.293	0.081	
	Clerical support workers (*n* = 26)	0.270	0.069		0.080	0.023		0.350	0.085		0.220	0.088		0.291	0.092	
	Elementary occupations (*n* = 15)	0.314	0.120		0.084	0.019		0.398	0.129		0.235	0.106		0.336	0.137	
	Service and sales workers (*n* = 48)	0.254	0.093		0.082	0.022		0.336	0.105		0.204	0.091		0.273	0.098	
	Skilled agricultural/Plant operators, etc. (*n* = 5)	0.272	0.025		0.082	0.015		0.353	0.030		0.166	0.052		0.266	0.050	
	Craft and related trades workers (*n* = 10)	0.303	0.074		0.089	0.011		0.392	0.078		0.256	0.113		0.339	0.109	
	Self‐employed (*n* = 15)	0.228	0.072		0.075	0.020		0.303	0.070		0.170	0.075		0.241	0.087	
	Housewife (*n* = 47)	0.250	0.092		0.097	0.063		0.346	0.086		0.201	0.078		0.276	0.086	
	Student (*n* = 21)	0.282	0.072		0.079	0.018		0.361	0.082		0.273	0.076		0.322	0.079	
	Unemployed/Retired/Unknown (*n* = 11)	0.299	0.110		0.082	0.013		0.381	0.110		0.250	0.128		0.326	0.123	

**TABLE 3:** Results of the relative Cutometer parameters, by demographic profile.

*N* = 298		*U*a/*U*f			*U*r/*U*e			*U*v/*U*e			*U*r/*U*f		
		Mean	SD	*p* value	Mean	SD	*p* value	Mean	SD	*p* value	Mean	SD	*p* value
Gender	Male (*n* = 49)	0.772	0.159	0.059	0.759	0.190	0.178	0.480	0.260	<0.001	0.531	0.166	0.001
	Female (*n* = 249)	0.818	0.108		0.799	0.187		0.311	0.102		0.614	0.153	
Age	18–25 (*n* = 48)	0.890	0.070	<0.001	0.945	0.107	<0.001	0.302	0.116	0.094	0.730	0.100	<0.001
	26–35 (*n* = 93)	0.831	0.101		0.834	0.148		0.321	0.171		0.641	0.132	
	36–45 (*n* = 85)	0.802	0.117		0.795	0.176		0.367	0.166		0.590	0.142	
	46–55 (*n* = 43)	0.767	0.118		0.670	0.171		0.344	0.102		0.501	0.136	
	56+ (*n* = 29)	0.700	0.139		0.582	0.181		0.367	0.165		0.436	0.156	
Phototype	II+III (*n* = 33)	0.838	0.087	0.035	0.784	0.204	0.376	0.366	0.121	0.391	0.577	0.155	0.232
	IV (*n* = 142)	0.792	0.123		0.779	0.197		0.343	0.183		0.591	0.168	
	V+VI (*n* = 123)	0.824	0.120		0.810	0.172		0.327	0.122		0.619	0.145	
Ethnicity	Caucasian (*n* = 8)	0.829	0.076	0.175	0.711	0.186	0.613	0.357	0.179	0.322	0.524	0.116	0.404
	Indian (*n* = 85)	0.787	0.128		0.787	0.184		0.350	0.206		0.596	0.167	
	Asian (*n* = 36)	0.831	0.115		0.790	0.214		0.371	0.145		0.584	0.173	
	Mixed race (*n* = 169)	0.816	0.116		0.800	0.185		0.325	0.120		0.610	0.151	
Skin type on the face	Dry (*n* = 43)	0.790	0.126	0.236	0.757	0.218	0.193	0.342	0.144	0.864	0.572	0.172	0.196
	Non‐dry (*n*=255)	0.814	0.118		0.798	0.182		0.338	0.156		0.606	0.155	
Skin type on the body	Dry (*n*=97)	0.806	0.116	0.665	0.795	0.187	0.856	0.358	0.162	0.141	0.593	0.149	0.565
	Non‐dry (*n* = 201)	0.812	0.121		0.791	0.189		0.330	0.149		0.604	0.162	
BMI	Healthy/underweight (*n* = 129)	0.824	0.116	0.196	0.807	0.189	0.485	0.318	0.117	0.056	0.619	0.156	0.209
	Overweight (*n* = 102)	0.805	0.110		0.782	0.180		0.342	0.144		0.591	0.152	
	Obese (*n* = 67)	0.793	0.135		0.779	0.199		0.374	0.215		0.580	0.168	
Occupation	Professionals (*n* = 48)	0.818	0.114	0.087	0.790	0.172	0.003	0.344	0.172	0.651	0.599	0.157	0.003
	Technicians and associate professionals (*n* = 44)	0.793	0.105		0.800	0.170		0.346	0.131		0.598	0.131	
	Managers (*n* = 8)	0.826	0.089		0.801	0.201		0.321	0.052		0.608	0.158	
	Clerical support workers (*n* = 26)	0.820	0.131		0.798	0.206		0.305	0.071		0.613	0.165	
	Elementary occupations (*n* = 15)	0.824	0.105		0.734	0.159		0.301	0.130		0.574	0.142	
	Service and sales workers (*n* = 48)	0.795	0.148		0.784	0.203		0.364	0.166		0.587	0.171	
	Skilled agricultural/Plant operators, etc. (*n* = 5)	0.748	0.095		0.603	0.147		0.302	0.060		0.467	0.132	
	Craft and related trades workers (*n* =10)	0.849	0.127		0.816	0.192		0.311	0.086		0.629	0.169	
	Self‐employed (*n* = 15)	0.775	0.123		0.730	0.154		0.397	0.320		0.542	0.153	
	Housewife (*n* = 47)	0.790	0.118		0.763	0.200		0.347	0.126		0.575	0.155	
	Student (*n* = 21)	0.890	0.063		0.967	0.114		0.290	0.079		0.753	0.103	
	Unemployed/Retired/Unknown (*n* = 11)	0.837	0.097		0.806	0.204		0.340	0.253		0.617	0.184	

Except *U*v (intermediate recovery), all absolute parameters were significantly influenced by gender. The average of *U*e (immediate deformation), *U*f (final distention), *U*r (immediate relaxation) and *U*a (total recovery) were generally higher for females compared to males (*U*e: 0.276 ± 0.082 vs. 0.209 ± 0.091, *p* < 0.001; *U*f: 0.360 ± 0.089 vs. 0.291 ± 0.098, *p* < 0.001; *U*r: 0.225 ± 0.086 vs. 0.162 ± 0.085, *p* < 0.001; *U*a: 0.297 ± 0.091 vs. 0.232 ± 0.101, *p* < 0.001). Compared to female subjects, *U*v/*U*e (ratio of viscoelastic to elastic extension) was significantly higher for males (0.480 ± 0.260 vs. 0.311 ± 0.102, *p* < 0.001), whereas *U*r/*U*f (ratio of elastic recovery to total deformation) was significantly lower (0.531 ± 0.166 vs. 0.614 ± 0.153, *p* = 0.001).

The absolute parameters (except *U*v) also differed significantly by age category; the mean values reduced systematically when shifting to older age categories. Compared to the top‐most category (ages 56+), the Cutometer readings for the youngest age group (ages 18–25) differed from 0.332 ± 0.099 to 0.228 ± 0.079 in terms of *U*a (*p* < 0.001), from 0.290 ± 0.089 to 0.240 ± 0.078 for *U*e (*p =* 0.001), 0.370 ± 0.097 to 0.319 ± 0.091 for *U*f (*p* = 0.013) and 0.273 ± 0.086 to 0.143 ± 0.068 for *U*r (*p* < 0.001). Skin recovery, whether expressed as a proportion of total deformation (*U*a/*U*f), pure elasticity (*U*r/*U*e) or *U*r/*U*f, was significantly lower among older subjects.

Compared to subjects of fairer phototypes (II and III), the facial skin for subjects in the darker group showed more important levels of immediate deformation (*U*e: 0.286 ± 0.093 vs. 0.232 ± 0.058, *p* = 0.001) and total levels of distortion (*U*f: 0.375 ± 0.101 vs. 0.312 ± 0.065, *p* < 0.001), as well as more important levels of recovery, both at immediate (*U*r: 0.237 ± 0.089 vs. 0.180 ± 0.063, *p* < 0.001) and final stages (*U*a: 0.313 ± 0.101 vs. 0.261 ± 0.059, *p* < 0.001). But the relative parameters generally did not reveal significant differences, except for *U*a/*U*f (0.838 ± 0.087 for first category [II–III] vs. 0.824 ± 0.120 for last category [V–VI], *p* = 0.035).

Ethnic wise, the absolute parameters were also significantly different while comparing the four (4) ethnic groups. The Cutometer readings for the volunteers categorised as mixed race appeared to distort (and return to normal state as well) by larger magnitude compared to the other groups altogether (Caucasian, Indian and Asian), both at immediate and thus total levels.

No significant patterns could be identified through skin type on the face (dry/non‐dry) and BMI, even if the outcome tended to be marginally more favourable for subjects with non‐dry skin, and the effect of BMI was limit significant (*p* = 0.056) when looking at *U*v/*U*e.

When subjects were categorised according to their occupation, significant differences were noted in terms of *U*r, *U*r/*U*e and *U*r/*U*f. A closer inspection shows that those categorised as skilled agricultural/plant workers and self‐employed had lower values for immediate recovery (*U*r), and thus naturally, smaller such rates when expressed as a proportion of immediate deformation (*U*r/*U*e) or total deformation (*U*r/*U*f).

### 3.2 Impact of Lifestyle Indicators

From Table 4, level of physical activity, nutritional behaviours and general state of health did not reveal salient differences in terms of Cutometer readings. Those who reported higher levels of fatigue had unexpectedly higher rates of recovery when expressed as a proportion of deformation (*U*a/*U*f, *U*r/*U*e and *U*v/*U*e).

**TABLE 4:** Results of absolute and relative Cutometer parameters, by lifestyle indicators.

*N* = 298			*U*e	*U*v	*U*f	*U*r	*U*a	*U*a/*U*f	*U*r/*U*e	*U*v/*U*e	*U*r/*U*f
Physical activity	Low physical activity (*n* =135)	Mean	0.264	0.084	0.348	0.217	0.288	0.813	0.797	0.345	0.604
		SD	0.088	0.032	0.094	0.094	0.097	0.124	0.199	0.172	0.17
	Moderate to intense physical activity (*n* =163)	Mean	0.266	0.084	0.349	0.212	0.286	0.808	0.789	0.334	0.598
		SD	0.086	0.031	0.094	0.084	0.095	0.115	0.179	0.137	0.147
	*p* value		0.856	0.96	0.881	0.639	0.834	0.747	0.726	0.55	0.714
Nutrition	Poor nutrition (*n* =113)	Mean	0.265	0.086	0.351	0.218	0.289	0.809	0.804	0.347	0.606
		SD	0.091	0.034	0.097	0.09	0.099	0.116	0.183	0.149	0.151
	Adequate nutrition (*n* =185)	Mean	0.265	0.082	0.347	0.212	0.285	0.811	0.785	0.334	0.598
		SD	0.085	0.03	0.092	0.088	0.094	0.121	0.191	0.157	0.162
	*p* value		0.979	0.338	0.732	0.562	0.703	0.874	0.411	0.492	0.68
General health	Poor health (*n* =159)	Mean	0.27	0.085	0.355	0.22	0.293	0.808	0.789	0.343	0.597
		SD	0.093	0.031	0.101	0.096	0.106	0.127	0.187	0.166	0.159
	Adequate health (*n* =139)	Mean	0.259	0.083	0.341	0.209	0.279	0.812	0.796	0.334	0.604
		SD	0.079	0.033	0.086	0.079	0.082	0.109	0.189	0.139	0.158
	*p* value		0.273	0.553	0.223	0.276	0.208	0.761	0.742	0.618	0.7
Fatigue	Extreme fatigue (*n* =103)	Mean	0.273	0.086	0.358	0.236	0.305	0.84	0.837	0.311	0.645
		SD	0.094	0.046	0.095	0.089	0.099	0.105	0.17	0.117	0.142
	Low fatigue (*n* =195)	Mean	0.261	0.083	0.343	0.203	0.277	0.794	0.769	0.353	0.577
		SD	0.083	0.021	0.093	0.087	0.093	0.123	0.193	0.168	0.161
	*p* value		0.251	0.468	0.192	0.002	0.014	0.002	0.002	0.027	<0.001
Sleep quality	Poor (*n* = 22)	Mean	0.292	0.083	0.375	0.246	0.311	0.819	0.831	0.305	0.646
		SD	0.083	0.021	0.091	0.101	0.1	0.132	0.22	0.116	0.191
	Fair (*n* =105)	Mean	0.269	0.084	0.354	0.224	0.297	0.827	0.814	0.327	0.621
		SD	0.085	0.035	0.092	0.084	0.093	0.107	0.164	0.139	0.141
	Good (*n* =171)	Mean	0.258	0.083	0.342	0.205	0.277	0.799	0.774	0.35	0.582
		SD	0.088	0.031	0.096	0.089	0.096	0.124	0.196	0.166	0.161
	*p* value		0.185	0.96	0.232	0.049	0.121	0.147	0.145	0.263	0.052
Transportation	Car or private vehicle (*n* =112)	Mean	0.248	0.082	0.33	0.196	0.267	0.794	0.774	0.37	0.575
		SD	0.077	0.02	0.084	0.085	0.091	0.128	0.191	0.184	0.162
	Public transportation (*n* =151)	Mean	0.273	0.085	0.358	0.225	0.295	0.817	0.801	0.318	0.615
		SD	0.09	0.04	0.096	0.089	0.092	0.111	0.186	0.132	0.156
	Bicycle/Motorcycle (*n* =10)	Mean	0.211	0.08	0.291	0.14	0.214	0.732	0.674	0.41	0.479
		SD	0.065	0.022	0.077	0.058	0.069	0.146	0.234	0.157	0.164
	Walking (*n* = 21)	Mean	0.329	0.088	0.418	0.283	0.372	0.889	0.875	0.277	0.685
		SD	0.09	0.02	0.103	0.066	0.098	0.069	0.12	0.066	0.09
	*p* value		<0.001	0.813	<0.001	<0.001	<0.001	0.001	0.024	0.004	0.001

Participants from the middle category of sleep quality (fair) generally had more favourable results in terms of the relative parameters when compared to those categorised as “good” and “poor”, but these results were not statistically supported in a significant manner.

Participants who used public transportation or walked to work had higher levels of deformation compared to those who travelled by car or bicycle/motorcyle (*U*e: 0.273 ± 0.090 and 0.329 ± 0.090 vs. 0.248 ± 0.077 and 0.211 ± 0.065, *p* < 0.001, *U*f: 0.358 ± 0.096 and 0.418 ± 0.103 vs. 0.330 ± 0.084 and 0.291 ± 0.077, *p* < 0.001). Yet, the recovery rates for those who used public transportation or walked to work were higher in terms of *U*a/*U*f (0.817 ± 0.111 and 0.889 ± 0.069 vs. 0.794 ± 0.128 and 0.732 ± 0.146, *p* = 0.001), *U*r/*U*e (0.801 ± 0.186 and 0.875 ± 0.120 vs. 0.774 ± 0.191 and 0.674 ± 0.234, *p* = 0.024), and *U*r/*U*f (0.615 ± 0.156 and 0.685 ± 0.090 vs. 0.575 ± 0.162 and 0.479 ± 0.164, *p* = 0.001), but lower in terms of Uv/Ue (0.318 ± 0.132 and 0.277 ± 0.066 versus 0.370 ± 0.184 and 0.410 ± 0.157, *p* = 0.004).

## 4 Discussion

This study sheds light on salient differences between subjects from various sociodemographic characteristics and lifestyle. The diversity of the population included and analysed as part of this investigation, arising naturally by its design and place of conduct, allows for a comparison of subgroups that is previously undocumented in the literature. It produces benchmark values that could be used as basis for clinical study designs and formal sample size calculations.

Aligned with the literature [28, 29, 30], the Cutometer measurements confirmed its capacity at sharply discriminating between age groups, consolidating the possibility of adopting such instruments for clinical trials investigating skin ageing features.

Attempting to unmask the blurred and contradictory nature of gender effects from the literature is not straightforward; skin deformation occurs to a more important extent for females compared to males, naturally matching also higher extents of recovery. But the ratio of viscoelastic recovery to elastic extension is higher for males, whereas the ratio of elastic recovery to total deformation is higher for females.

Distortions and recovery were both significantly higher in subjects of phototype V and VI compared to the fairer groups, thus without much impacting the relative parameters, the only significant difference noted in terms of *U*a/*U*f. Disregarding the phototype subgroup II+III due to a smaller sample size (*n* = 33), subjects of darker phototypes had higher potency to return to a normal state following a distortion, and thus more desirable features. This matches closely with the findings from a previous study [31], which argue that elasticity is affected by photodamage arising from UV exposure, thus affecting mostly subjects of fairer phototype. The extent of UV damage is also dependent on melanin levels [32], the latter naturally more prominent among individuals of darker skin colour [33]. This feature limits the extent of UV‐induced collagen degradation within the same population, allowing to preserve skin elasticity for longer durations. Increased collagen density is also an inherent characteristic among individuals bearing darker skin colour [34], contributing to enhanced skin firmness. In the present study, we also report that subjects categorised as having a mixed race had more desirable features in terms of elasticity.

While the effect of BMI from the literature was still unclear [19, 20], this study also did not find conclusive evidence of difference between subjects from various BMI categories. An interesting pattern (*p* value = 0.056) in the ratio of viscoelastic to elastic extension (*U*v/*U*e) is reported. Although normally this parameter gets closer to the value of one (1) in older subjects, and thus, higher values would typically characterise an undesirable feature, this same parameter should be interpreted differently and with care while dealing with subjects from various BMI backgrounds.

This paper also investigated the effect of occupation per se on facial skin ageing; the literature so far mostly focused on the extent of sun exposure [35, 36]. There is a clear effect of occupation in the sense that subjects involved in elementary occupations, working as skilled agricultural/plant operators, or categorising as self‐employed or housewives, had reduced elastic features compared to the subjects in other categories. Along the same lines, those who walked or used public transportation to work had higher levels of distortion with limited recovery compared to those traveling by car or bicycle/motorcyle. The obvious underlying explanation is the extent of exposure associated to the sun that these tasks generally entail, but there is a need to recognise possible confounders among reasons leading to skin ageing.

Nutritional behaviours and physical activity did not impact the skin biomechanical properties, whereas the level of fatigue revealed an unexpected pattern. The paper stresses on the importance of just enough sleep, as the elastic features within this category were convincingly more favourable.

This study and its findings recognise the need to cater for the demographic profile and lifestyle of the individuals while deciding among various treatment alternatives targeted at improving skin elasticity. The degree of and nature of any distorted skin ageing feature is unlikely to be similar across various subgroups, requiring scrutiny while choosing among interventions, including formulations, dosage and duration. The importance of handling skin degradation in a personalised manner is inherently implied.

Moreover, clinical trials aimed at assessing the effect of anti‐ageing formulations should take into consideration those demographic intricacies, whether by design, statistical adjustments, or both. Failure to do so may lead to over‐ or underestimation of treatment effects due to the populations considered, or imbalances between groups in terms of subject characteristics.

The limitation of the study is that subject recruitment was done on a convenience, first‐come first‐recruited basis, naturally causing some imbalance in terms of sample size when comparing subgroups. But on most occasions, the sample sizes were sufficiently large, and the statistical procedures sufficiently empowered to detect meaningful differences between groups. Additionally, the study designed as monocentric implicitly limits a direct generalisation of results to other populations. But the geographical location of the institution within which the study was conducted was reasonably conducive for the enrolment of a population that is historically diverse, and thus reducing the impact of any bias.

The cross‐sectional nature of this study relies on data collected at a single timepoint, not offering the possibility to report long‐term lifestyle influences on skin ageing features. This includes the use of skin ageing products, including topical or oral vitamin and antioxidant supplementation. Future longitudinal studies may address exploring this avenue, which will allow further understanding of the variations in skin elasticity due to lifestyle.

## 5 Conclusions

Demographic background and lifestyle inevitably have an impact on facial skin ageing and subsequently, the instrumental measurements capturing these features are affected. This is evidenced through the significant differences revealed in this paper. There is a need to recognise such differences while designing clinical trials, including during the interpretation of the results generated thereof. The findings also strengthen the need for a personalised approach while deciding among treatment interventions for skin ageing–related features.

## Conflicts of Interest

The authors declare no conflicts of interest.

## Data Availability

The data used for this study can be requested from the corresponding author for reasonable purposes.
